# The melanoma brain metastatic microenvironment: aldolase C partakes in shaping the malignant phenotype of melanoma cells – a case of inter‐tumor heterogeneity

**DOI:** 10.1002/1878-0261.12872

**Published:** 2020-12-14

**Authors:** Sivan Izraely, Shlomit Ben‐Menachem, Orit Sagi‐Assif, Tsipi Meshel, Sapir Malka, Alona Telerman, Matias A. Bustos, Romela Irene Ramos, Metsada Pasmanik‐Chor, Dave S. B. Hoon, Isaac P. Witz

**Affiliations:** ^1^ The Shmunis School of Biomedicine and Cancer Research George S. Wise Faculty of Life Science Tel Aviv University Israel; ^2^ Department of Translational Molecular Medicine John Wayne Cancer Institute at Providence Saint John's Health Center Santa Monica CA USA; ^3^ Bioinformatics Unit The George S. Wise Faculty of Life Science Tel Aviv University Israel

**Keywords:** aldolase C, brain metastasis, melanoma, microglia, tumor microenvironment

## Abstract

Previous studies indicated that microglia cells upregulate the expression of aldolase C (ALDOC) in melanoma cells. The present study using brain‐metastasizing variants from three human melanomas explores the functional role of ALDOC in the formation and maintenance of melanoma brain metastasis (MBM). ALDOC overexpression impacted differentially the malignant phenotype of these three variants. In the first variant, ALDOC overexpression promoted cell viability, adhesion to and transmigration through a layer of brain endothelial cells, and amplified brain micrometastasis formation. The cross‐talk between this MBM variant and microglia cells promoted the proliferation and migration of the latter cells. In sharp contrast, ALDOC overexpression in the second brain‐metastasizing melanoma variant reduced or did not affect the same malignancy features. In the third melanoma variant, ALDOC overexpression augmented certain characteristics of malignancy and reduced others. The analysis of biological functions and disease pathways in the ALDOC overexpressing variants clearly indicated that ALDOC induced the expression of tumor progression promoting genes in the first variant and antitumor progression properties in the second variant. Overall, these results accentuate the complex microenvironment interactions between microglia cells and MBM, and the functional impact of intertumor heterogeneity. Since intertumor heterogeneity imposes a challenge in the planning of cancer treatment, we propose to employ the functional response of tumors with an identical histology, to a particular drug or the molecular signature of this response, as a predictive indicator of response/nonresponse to this drug.

## Graphical abstract

The melanoma‐microglia cross‐talk upregulates the expression of aldolase C (ALDOC) in melanoma cells from several patients. The response of melanoma cells from 2 different patients to ALDOC upregulation was diametrically divergent. Whereas several metastasis‐associated functions including brain metastasis formation were augmented in one melanoma cell line (Mel 1), these functions were attenuated in the other melanoma cell line (Mel 2).

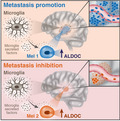

**Abbreviations**
ALDOC: aldolase C
BBB: blood–brain barrier
BEC: brain endothelial cells
CM: conditioned medium
CNS: central nervous system
ECM: extracellular matrix
MBM: melanoma brain metastasis
MCM: melanoma‐conditioned medium
MGCM: microglia‐conditioned medium
MMP‐2: matrix metalloproteinase‐2
RS9: ribosomal protein S9
TEM: transendothelial migration
β2m: β2 microglobulin

## 1 Introduction

Of all noncentral nervous system (CNS) solid tumors, cutaneous melanoma has one of the highest prevalences of brain metastasis. Forty to fifty percent of advanced‐stage melanoma patients are diagnosed with melanoma brain metastasis (MBM) [1]. The prognosis of such patients is poor, and their response to novel modalities of therapy is limited [2].

To discover new targets for therapy, efforts should be made to comprehend melanoma metastasis pathogenesis and the mechanisms underlying their formation [3]. Once melanoma cells migrate to the brain, they interact with brain microenvironmental cells such as endothelial cells, astrocytes, and microglia [4, 5]. Such interactions may either lead to successful metastatic progression or to the elimination of early stages of metastasis [4, 5, 6, 7]. Revealing the pathways leading to metastatic progression or regression is crucial for the identification of novel therapeutic targets and treatment modalities.

As previously demonstrated [4, 8], melanoma and microglia cells interact in a bidirectional, reciprocal manner. Melanoma cells can exert morphological changes in microglia cells, enhancing their proliferation, migratory capacity, and matrix metalloproteinase‐2 (MMP‐2) activation. Reciprocating microglia cells induce phenotypic changes in melanoma cells increasing their malignant phenotype. Microglia cells promote melanoma cell proliferation, migration, ability to penetrate the brain endothelium, ability to grow as spheroids in 3D cultures, and MMP‐2 activity. Both cells induce alterations in gene expression profiles, cell signaling, and cytokine secretion in the reciprocal cell type. For instance, RNA sequencing (RNA‐seq) analysis revealed that microglia‐secreted factors upregulate the expression levels of aldolase C (ALDOC) in MBM cell lines [4].

ALDOC, a member of the aldolase family, is a glycolytic enzyme, expressed primarily in the brain, for example, by astrocytes and neurons [9, 10]. ALDOC is responsible for catalyzing the reversible conversion of fructose‐1,6‐bisphosphate to glyceraldehyde‐3‐phosphate and dihydroxyacetone phosphate. Additionally, ALDOC can be involved in nonglycolytic roles and in cancer progression [9, 11]. ALDOC is highly expressed by several types of metastatic cancers such as lung and gastric cancer, adenocarcinoma, and gallbladder carcinoma [12, 13, 14, 15]. In the latter, ALDOC promotes glucose uptake and consequently proliferation [13]. In contrast to these findings, ALDOC is positively correlated with longer survival and good prognosis of glioblastoma patients and can inhibit cell migration and invasion in glioblastoma and additional cancer types [16]. ALDOA and ALDOB, the other members of the aldolase family, are involved in colon cancer progression and are associated with poor prognosis [17, 18].

The aim of the present study was to reveal the functional role of ALDOC in shaping the malignant phenotype of MBM. This was done by overexpressing ALDOC in melanoma cells and evaluating molecular and functional malignancy parameters of these cells.

## 2 Materials and methods

### 2.1 Cell culture

Cutaneous melanoma variants (YDFR.C, DP.C, M12.C, and M16.C) and MBM variants (YDFR.CB3, DP.CB2, M12.CB3, and M16.CB3) were established from the parental cell lines YDFR (kindly provided by M. Micksche, Department of Applied and Experimental Oncology, Vienna University, Austria), DP‐0574‐Me, UCLA‐SO‐M12, and UCLA‐SO‐M16 (kindly provided by D. S. B. Hoon) [19, 20]. In short, human brain metastatic melanoma cells were inoculated subdermally into nude mice to establish the cutaneous (C) variants. Cells from these cultures were inoculated intracardially and passaged in the brain for two or three passages yielding the brain metastatic variants (CB2 or CB3, respectively). Melanoma cells were maintained in RPMI‐1640 medium (Biological Industries, Kibbutz Beit Haemek, Israel) supplemented with 10% fetal bovine serum (FBS), 2 mmol·mL^−1^ l‐glutamine, 100 units·mL^−1^ penicillin, 0.1 mg·mL^−1^ streptomycin, and 12.5 units·mL^−1^ nystatin. Immortalized human microglia‐SV40 cells (ABM, Milton, ON, Canada) were maintained on 100 μg·mL^−1^ collagen I, rat tail (BD Biosciences, Bedford, MA, USA) in PriGrow III Medium (ABM) supplemented with 10% FBS, 2 mmol·mL^−1^ l‐glutamine, 100 units·mL^−1^ penicillin, 0.1 mg·mL^−1^ streptomycin, and 0.00025 units·mL^−1^ amphotericin B. Immortalized human brain microvascular endothelial cells (hCMEC/D3), derived from human temporal lobe microvessels, were kindly provided by C. Nahmias and P.‐O. Couraud (Inserm, U1016; Institute Cochin, Paris, France) and were maintained (until passage 35) on 100 μg·mL^−1^ collagen I, rat tail in EBM‐2 medium (Clonetics; Cambrex BioScience, Wokingham, UK) supplemented with 5% FBS, 2 mmol·mL^−1^ l‐glutamine, 100 units·mL^−1^ penicillin, 100 units·mL^−1^ streptomycin, 12.5 units·mL^−1^ nystatin, 0.01 ng·mL^−1^ basic FGF, 0.025 ng·mL^−1^ ascorbic acid, 10 mm HEPES‐buffered saline, and 0.002 ng·mL^−1^ hydrocortisone. Human embryonic kidney 293T cell line was maintained in DMEM supplemented with 10% FBS, 2 mmol·mL^−1^ l‐glutamine, 100 units·mL^−1^ penicillin, 0.1 mg·mL^−1^ streptomycin, and 12.5 units·mL^−1^ nystatin. 0.5% FBS supplemented medium was used for starvation in all the experiments. Cells were routinely cultured in an incubator with humidified air with 5% CO_2_ at 37 °C.

### 2.2 Preparation of melanoma or microglia‐conditioned medium

Melanoma or microglia cells were cultured for 24 h and then starved for 24 h. Melanoma‐conditioned medium (MCM) or microglia‐conditioned medium (MGCM) was collected, centrifuged for 5 min at 427 RCF, and filtered (0.45 μm; Whatman GmbH, Dassel, Germany).

### 2.3 3D bulk cultures for RNA extraction

Melanoma cell 3D spheres for RNA extraction were formed as previously described [21]. Briefly, melanoma cell suspension was mixed 1 : 4 with Matrigel and then cultured in Millicell hanging cell culture inserts with 1.0‐µm PET transparent membranes (Merck Millipore, Darmstadt, Germany) on 6‐well plates. Membranes were precoated with 1 : 1 Matrigel/medium solution and incubated at 37 °C for 1 h.

Microglia cells were seeded in 6‐well microplates. Control wells contained only medium. 3D spheroids were transferred into 15‐mL falcon tubes, mixed with 9 mL of 5 mm EDTA in cold 1×PBS, and incubated on a rocker for 45 min to detach from the Matrigel. Spheroids were washed twice with cold physiological 1×PBS and sedimented by centrifugation at 427 RCF. Cell pellet was used for RNA extraction and RT–qPCR.

### 2.4 Construction of the expression vector and stable overexpression of ALDOC

The overexpression construct of human *ALDOC* (NM_005165) was created by PCR amplification of genomic DNA by Phusion^®^ High‐Fidelity DNA Polymerase (Thermo Fisher Scientific, Bedford, MA, USA), using the following primers (designed based on the GenBank Nucleotide Database of the NCBI website): ALDOC: S‐5′‐CTCTCTCCGCGGTCACCATGCCTCACT‐3′, AS‐5′‐CTGCTGTTAATTAATCAGTAGGCATGGTTGGCAA‐3′. The generated fragment was digested with *Sac2* and *PacI* and ligated into the corresponding sites of pCMV‐Neo vector (Clontech Laboratories, Inc., Mountain View, CA, USA). PCR products of *ALDOC* were sequenced and found to be identical to the published sequence. Production of plasmid and viral vectors and melanoma transfection was performed as previously described [22]. To produce mCherry‐expressing cells, melanoma cells were similarly transduced with a pQCXIP‐*mCherry* plasmid and were selected using 2 µg·mL^−1^ puromycin (InvivoGen, San Diego, CA, USA).

### 2.5 ALDOC downregulation in melanoma cells

The downregulation of ALDOC was established as previously described [19] using a mixture of three different pGIPZ vectors containing shRNA sequences targeting ALDOC mRNA (NM_005165) (RHS4430‐200293158, RHS4430‐200297123, and RHS4430‐200290935; Dharmacon, Lafayette, CO, USA). A sh‐nonsilencing pGIPZ vector (RHS4531) was used as a negative control.

### 2.6 Aldolase activity assay

Melanoma cells were analyzed for aldolase activity using the Aldolase Activity Assay Kit (Colorimetric; Abcam, Cambridge, UK) according to the manufacturer's instructions.

### 2.7 RNA preparation and reverse transcription quantitative real‐time PCR (RT‐qPCR)

Total cellular RNA was extracted using EZ‐RNA Total RNA Isolation Kit (Biological Industries). RNA concentrations were determined by the absorbance at 260 nm, and quality control standards were A260/A280 = 1.8–2.0. RNA samples were used for cDNA synthesis using the qScript cDNA Synthesis Kit (Quantabio, Beverly, MA, USA) according to the manufacturer's instructions. For mRNA amplification, primers were designed based on the GenBank Nucleotide Database of the NCBI website: ALDOC: S‐5′‐TGCCTATTGTGGAACCTGAA‐3′, AS‐5′‐ACAGCAGCCAAGACCTTCTC‐3′; RS9: S‐5′‐CGGAGACCCTTCGAGAAATCT‐3′, AS‐5′‐GCCCATACTCGCCGATCA‐3′; human β2m: S‐5′‐ATGTAAGCAGCATCATGGAG‐3′, AS‐5′‐AAGCAAGCAGAATTTGGAAT‐3′; and mouse β2m: S‐5′‐CTGGTCTTTCTGGTGCTTGT‐3′, AS‐5′‐GCGTGAGTATACTTGAATTTGAG‐3′.

Amplification reactions were performed with SYBR Green I (Thermo Fisher Scientific) in triplicates in a Rotor‐Gene 6000TM Thermal Cycler (Corbett Life Science, Mortlake, Vic., Australia). PCR amplification was performed over 35–40 cycles (95 °C for 15 s, 59 °C for 20 s, and 72 °C for 15 s).

### 2.8 Western blotting

For protein expression analysis, lysate preparations were performed as previously described [19]. Proteins were separated on 4–12% Bis/Tris gels (Thermo Fisher Scientific) and transferred onto nitrocellulose membranes. The membranes were blocked at RT with 3% BSA diluted in TBS/Tween for 1 h. The following primary Abs were used: aldolase C antibody (Ab) (N‐14) (1 : 500; Santa Cruz Biotechnology, Santa Cruz, CA, USA) and anti‐beta tubulin Ab (1 : 500; Abcam). Horseradish peroxidase‐conjugated donkey anti‐goat Ab or goat anti‐rabbit Ab (1 : 10 000; Jackson ImmunoResearch Laboratories, West Grove, PA, USA) was used as secondary Abs. The gel bands were visualized by chemiluminescence ECL reactions (Merck Millipore).

### 2.9 Flow cytometry

Human ICAM‐1/CD54 Ab (0.5 µg; R&D Systems, Minneapolis, MA, USA) followed by a secondary FITC‐conjugated goat anti‐mouse Ab (1 : 50; Jackson ImmunoResearch Laboratories) was used for ICAM‐1 detection using flow cytometry as previously described [22]. Antigen expression was determined using Flow Cytometer S100EXi (Stratedigm; San Jose, CA, USA) with cellcapture software (Stratedigm, Inc., San Jose, CA, USA) and flowjo v10 (BD biosciences, Ashland, OR, USA). Dead cells were gated out from the analysis.

### 2.10 Viability assay (XTT)

Cell viability was measured by Cell Proliferation Kit (XTT; Biological industries) used according to the manufacturer's instructions.

### 2.11 Migration through extracellular matrix

1 × 10^5^ melanoma cells were loaded onto collagen‐coated transwell inserts (8 μm; Corning Costar Corp., New York, NY, USA) and allowed to migrate for 24 h toward starvation medium. Alternatively, 1 × 10^5^ microglia cells were loaded similarly and were allowed to migrate toward 5 × 10^4^ melanoma cells seeded at the bottom of the well or toward melanoma‐conditioned medium (MCM). Migrating cell fixation and analysis were performed as previously described [19].

### 2.12 Adhesion to brain endothelial cells

Adhesion of melanoma cells to brain endothelial cells (BEC) was performed as previously described [22]. Adhesion of mCherry‐expressing cells was measured at wavelength of 590/645. To obtain the percentage of adherent cells, the optical density (OD) of the adherent cells was divided by the OD of the total cells plated.

### 2.13 Transendothelial migration through a blood–brain barrier model

Transendothelial migration (TEM) of mCherry‐expressing melanoma cells was performed as previously described [4].

### 2.14 Animals

Male athymic nude mice (BALB/c background) were purchased from Harlan Laboratories Limited (Jerusalem, Israel). The mice were housed and maintained in laminar flow cabinets under specific pathogen‐free conditions in the animal quarters of Tel Aviv University and in accordance with current regulations and standards of the Tel Aviv University Institutional Animal Care and Use Committee. The mice were used when they were 7–8 weeks old.

### 2.15 Orthotopic inoculation of tumor cells and tumorigenicity and metastasis formation assays

To generate subcutaneous tumors, mice were inoculated subcutaneously as previously described [20]. To test the tumorigenic properties of derived cell lines, subcutaneous tumors were measured once a week using a caliper. Tumor volume was obtained by the ellipsoid volume calculation formula Tumor volume = 0.5 × (length × width × width) [23]. Mice were put to death when tumors were larger than 2 cm in one of the measured dimensions, or when a more than 20% body weight loss occurred. Brains were harvested and immediately frozen and stored at −70 °C, until used for RNA extraction. Detection of human cells (micrometastases) in mouse brain by RT–qPCR was performed as previously described [20].

### 2.16 RNA sequencing analysis

RNA was extracted using miRNeasy Mini Kit (Qiagen, Valencia, CA, USA). Concentration of purified total RNA was measured using the Quant‐iT RiboGreen RNA Assay (Life Technologies, Carlsbad, CA, USA), and RNA quality was assessed by the RNA ScreenTape Assay on the Agilent TapeStation 2200 (Agilent Technologies, Santa Clara, CA, USA). Using 1 µg of high‐quality (RIN ≥ 7.0) total RNA, mRNA libraries were prepared with the NextFlex Rapid Directional mRNA‐Seq Kit (Bioo Scientific, Austin, TX, USA). Quality and quantity of final libraries were assessed by High Sensitivity D1000 Assay (Agilent Technologies) and Qubit dsDNA HS Assay (Life Technologies), respectively. Libraries were pooled and sequenced on an Illumina NextSeq 550 (Illumina Inc, San Diego, CA, USA) using 76‐bp paired‐end reads.

Raw RNA‐seq reads were checked for overall quality and filtered for adapter contamination using trimmomatic (version 0.36) [24]. The filtered reads were then mapped to the GENCODE comprehensive gene annotation reference set (version 19) using the star aligner (version 2.4.2a) [25] with default parameters. Read counts for each feature were generated using the ‘‐‐quantModeGeneCounts’ function in star. Significantly differentially expressed genes were identified using ANOVA with a significance threshold of fold change (FC) < −1.5 or FC > 1.5 and *P*‐value > 0.05.

### 2.17 Reverse‐phase protein array

Protein lysates from melanoma cells were extracted as previously described [19]. Reverse‐phase protein array (RPPA) analysis was performed by the RPPA Core Facility at the University of Texas, MD Anderson Cancer Center (G. Mills).

### 2.18 Bioinformatic analysis tools

Venny tool was used to compare between differentially expressed (DE) gene lists or protein lists (http://bioinfogp.cnb.csic.es/tools/venny/). Enriched biological processes of the DE genes were obtained through the analysis of disease and biofunctions (threshold, *z*‐score < −2 or *z*‐score > 2) using Qiagen Ingenuity Pathway Analysis (ipa, https://digitalinsights.qiagen.com/products‐overview/discovery‐insights‐portfolio/analysis‐and‐visualization/qiagen‐ipa/).

### 2.19 Biostatistic analysis

Data were analyzed using Student's *t*‐test and considered significant at *P*‐values ≤ 0.05. Bar graphs represent mean and standard error of the mean (SEM) across multiple independent experimental repeats.

## 3 Results

### 3.1 ALDOC is involved in shaping the malignant phenotype of certain melanoma cells

#### 3.1.1 Human microglia‐derived factors upregulate ALDOC expression by melanoma brain metastases

**Fig. 1:**
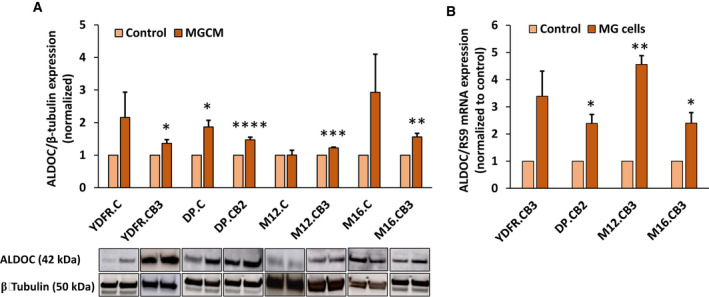
ALDOC expression in melanoma cells is upregulated by microglia‐conditioned medium. (A) Western blot was used to analyze the expression of aldolase C (ALDOC) protein in YDFR, DP, M12, and M16 cutaneous (C) or brain metastatic (CB2 or CB3) variants pretreated for 24 h with microglia‐conditioned medium (MGCM). The bars represent the relative expression of ALDOC normalized to β‐tubulin in melanoma cells incubated with MGCM, compared with untreated control cells + SEM obtained in three independent experiments. Significance was evaluated using Student's *t*‐test for MGCM‐treated melanoma cells, compared with untreated melanoma cells. *P < 0.05, **P < 0.01, ***P < 0.001, ****P < 0.0005. Representative blots are presented. (B) ALDOC expression in melanoma 3D spheroids cultured with or without microglia in 6‐well plates was determined using RT–qPCR. The bars represent the relative mRNA expression of ALDOC normalized to RS9, in 3D spheroids grown with microglia (MG), compared to control 3D spheroids grown without microglia + SEM obtained in three independent experiments. Significance was evaluated using Student's *t*‐test for melanoma cells grown with microglia, compared with melanoma cells alone. *P < 0.05, **P < 0.01.

We showed previously that MGCM regulates the expression of several molecules in human melanoma brain metastases (MBM) [4]. The glycolytic enzyme aldolase C (ALDOC) was among these upregulated genes. We investigated whether the MGCM‐mediated upregulation of ALDOC expression is restricted to MBMs being preconditioned by microglia‐derived signals or if matching cutaneous melanoma would also express upregulated levels of ALDOC. The results indicated that MGCM significantly upregulated ALDOC expression in four different MBM cell lines (YDFR.CB3, DP.CB2, M12.CB3, and M16.CB3), but only in one of the cutaneous variants (DP.C) (Fig. 1A). To mimic the *in vivo* setting, ALDOC expression was assessed in melanoma cells grown as 3D spheroids in co‐culture with human microglia cells. ALDOC mRNA expression was higher in 3D spheroids co‐cultured with microglia cells, than in respective 3D spheroids of melanoma cells cultured without microglia (Fig. 1B).

#### 3.1.2 ALDOC influence on melanoma cell viability is microenvironment‐dependent

Metastasis formation by cancer cells penetrating a distant organ depends on the ability of the disseminated cells to survive and proliferate in the new organ microenvironment [26, 27]. ALDOC is known to be involved in cell proliferation and growth [28]. We therefore examined the effect of ALDOC overexpression or downregulation on melanoma cell viability. To generate cell populations overexpressing ALDOC, MBM cells were infected with lentivirus containing ALDOC cDNA constructs or the mock plasmid as control cells. ALDOC overexpression increased aldolase enzymatic activity in YDFR.CB3 cells while it had no effect on aldolase enzymatic activity in DP.CB2 cells (data not shown). To downregulate endogenous ALDOC expression, MBM cells were infected with virions containing a short hairpin (sh) ALDOC‐pGIPZ plasmid (shALDOC) or sh‐nonsilencing pGIPZ vector to serve as controls (shControl).

**Fig. 2:**
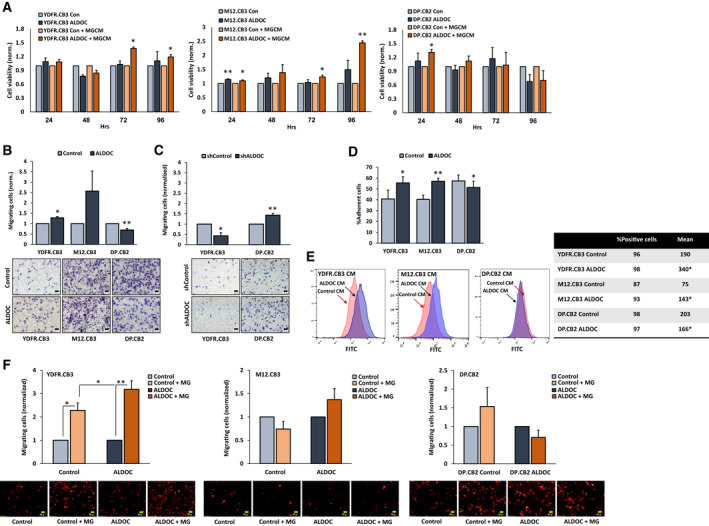
ALDOC differently shapes the malignant phenotype of melanoma cells. (A) Viability of ALDOC overexpressing or control melanoma cells incubated with microglia‐conditioned medium (MGCM) or starvation medium was measured by XTT. Subtraction of the absorbance at 450 and 630 nm was determined for each well. The bars represent the viability of melanoma cells at the indicated time point, normalized to control melanoma cells (with/without MGCM) + SEM in three independent experiments. Six replicates were performed in each experiment. Significance was evaluated using Student's *t*‐test for ALDOC overexpressing melanoma cells, compared with control melanoma cells. *P < 0.05, **P < 0.005. (B, C) Melanoma cells were allowed to migrate through collagen‐coated transwells for 24 h. The migrated cells were fixed and counted in 6–10 fields. The bars represent the number of migrating ALDOC overexpressing cells (B) or ALDOC knocked‐down cells (C) per field in three independent experiments performed in duplicates, normalized to control cells + SEM. Significance was evaluated using Student's *t*‐test for ALDOC overexpressing melanoma cells, compared with control melanoma cells. *P < 0.05, **P < 0.01. Representative images are shown (10× magnification, scale bar: 50 µm). (D) mCherry‐expressing melanoma cells were allowed to adhere to BECs for 30 min at 37 °C. The fluorescence signal of labeled cells was measured before and after removal of nonadherent cells. The bars represent the average % adherent cells + SEM in three independent experiments. Six replicates were performed in each experiment. Significance was evaluated using Student's *t*‐test for ALDOC overexpressing melanoma cells, compared with control melanoma cells. *P < 0.05, **P < 0.01. (E) BECs were treated with melanoma‐conditioned medium (MCM) of ALDOC overexpressing or control melanoma cells for 72 h. Representative flow cytometry histograms of ICAM‐1 expression in BEC treated with MCM of ALDOC overexpressing cells (blue) or control cells (red) are shown. Average % positive cells and mean expression obtained in three independent experiments are presented in the table. Significance was evaluated using Student's *t*‐test for ICAM‐1 mean expression in BEC treated with CM of ALDOC overexpressing cells, compared to BEC treated with CM of control cells. *P < 0.05. (F) TEM of mCherry‐expressing melanoma cells. The migrated cells were fixed and counted in 6–10 fields. The bars represent the number of control or ALDOC overexpressing melanoma cells migrating toward microglia cells (MG) normalized to the number of the same melanoma cells migrating toward starvation medium + SEM. Significance was evaluated using Student's *t*‐test for cells migrating toward microglia cells compared with cells migrating toward starvation medium, and for ALDOC overexpressing cells compared with control cells. *P < 0.05, **P < 0.01. Representative images are shown (10× magnification, scale bar: 50 µm).

XTT assays demonstrated that ALDOC expression levels did not alter melanoma viability in either YDFR.CB3 or DP.CB2 ALDOC overexpressing cells (Fig. 2A) or in cells expressing downregulated ALDOC levels (data is not shown). ALDOC overexpression slightly increased melanoma viability in M12.CB3 cells, only after a 24‐h cultivation. This effect disappeared in the following time points measured (48, 72, and 96 h). As described in Section 3.1.1, microglia‐secreted factors, as well as microglia cells, upregulated ALDOC expression in MBM. Previous results [4] demonstrated that microglia‐derived soluble factors support melanoma viability. We therefore asked whether ALDOC upregulation further contributes to melanoma viability in the presence of microglia‐derived soluble factors. To simulate such a scenario, we measured the effects of MGCM on the viability of melanoma cells expressing high levels of ALDOC. ALDOC overexpressing melanoma cells and control (pCMV‐infected) melanoma cells were treated with MGCM or starvation medium for 24, 48, 72, and 96 h, and cell viability was then determined using XTT assays. MGCM significantly increased the viability of ALDOC overexpressing YDFR.CB3 and M12.CB3 cells compared with control treated cells (Fig. 2A). This effect lasted for 96 h following exposure. However, the viability‐enhancing effect of MGCM on ALDOC overexpressing DP.CB2 cells lasted only 24 h. These results demonstrated that microglia‐mediated ALDOC upregulation in melanoma cells promotes melanoma viability, but only in the presence of microglia‐derived factors, contributing to the survival of MBM in the brain microenvironment.

#### 3.1.3 ALDOC regulates the migratory capacity of MBM cells

The ability to invade into surrounding tissues is a fundamental characteristic of metastatic melanoma cells [29]. As ALDOC interacts with cytoskeletal proteins (e.g., F‐actin, tubulin) [30] and alters the migratory capacity of tumor cells [9], we examined whether ALDOC overexpressing melanoma cells would manifest an altered ability to migrate through collagen‐coated transwells. ALDOC overexpressing YDFR.CB3 cells migrated more efficiently than the respective control cells (Fig. 2B). On the contrary, ALDOC reduced the migratory capacity of ALDOC overexpressing DP.CB2 cells. ALDOC did not have a significant impact on M12.CB3 migration.

To further confirm these results, we demonstrated that the downregulation of ALDOC significantly decreased the migratory capacity of YDFR.CB3 cells and increased the migration of DP.CB2 cells through collagen‐coated transwells (Fig. 2C). Thus, ALDOC influences the migration of these two MBM variants in an opposite manner, demonstrating that the same type of tumor cells, derived from different individual melanoma patients, may be differentially influenced by the same microenvironmental cue.

#### 3.1.4 ALDOC regulates the adhesion of melanoma cells to brain endothelial cells

For metastatic melanoma cells to invade the brain tissue, they have to penetrate the brain endothelium. An initial step in this process is the adhesion of melanoma cells to the brain endothelial layer [31]. In order to establish whether ALDOC regulates melanoma cell adhesion to BEC, we compared the adhesion of control and ALDOC overexpressing cells to BEC. ALDOC overexpressing YDFR.CB3 and M12.CB3 cells adhered significantly better to BEC than control cells (Fig. 2D). ALDOC overexpressing DP.CB2 cells adhered less than control cells to BECs.

In view of the important role played by endothelial ICAM‐1 in mediating the adhesion of melanoma cells to the endothelium [22, 32], ICAM‐1 expression was measured in BEC treated with MCM derived from ALDOC overexpressing MBM cells and in BEC that were treated with MCM of control MBM cells. As shown in Fig. 2E, supernatants of ALDOC overexpressing YDFR.CB3 and M12.CB3 cells significantly upregulated the expression of ICAM‐1 in BEC, whereas supernatants of ALDOC overexpressing DP.CB2 cells significantly downregulated the expression of ICAM‐1 in BEC. These results demonstrated again a differential response of the three different melanoma variants to ALDOC and suggested that the ALDOC‐mediated up‐ or downregulation of ICAM‐1 may underlie the increased or decreased adhesion of melanoma cell lines to BEC.

#### 3.1.5 ALDOC regulates melanoma cell transendothelial migration through BEC

We previously demonstrated that microglia cells have the capacity to chemo‐attract melanoma cells, thereby enhancing their ability to transmigrate through BEC [4]. In order to examine whether ALDOC upregulation in melanoma cells facilitates melanoma penetration of the blood–brain barrier (BBB), we used a BBB model, which serves to simulate the extravasation of melanoma cells and their migration toward microglia. ALDOC overexpression significantly enhanced the ability of mCherry‐labeled YDFR.CB3 cells to transmigrate through BEC toward microglia cells (Fig. 2F). However, it did not affect the capacity of M12.CB3 or DP.CB2 cell ability to penetrate through BEC. These results indicate that ALDOC is functionally involved in the migration and TEM of certain metastatic melanoma cells.

#### 3.1.6 ALDOC promotes melanoma brain micrometastasis formation

**Fig. 3:**
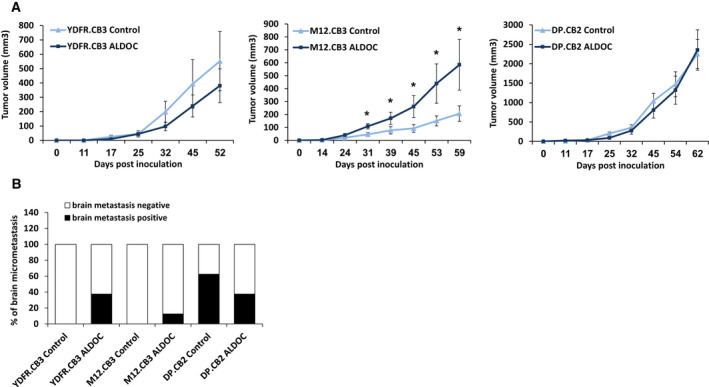
ALDOC promotes or inhibits melanoma brain metastasis formation of different melanoma cell variants. (A, B) BALB/c nude mice were subcutaneously inoculated with 1 × 10^6^ ALDOC overexpressing or control melanoma cells. (A) Tumor dimensions of cutaneous tumors were measured using a caliper, and volume was calculated as described in Section 2. The average tumor volume in mice in each group ± SEM is presented. Significance was evaluated using Student's *t*‐test for volume of tumors originating from ALDOC overexpressing melanoma cells, compared with those originating from control melanoma cells. *P < 0.05. (B) RT–qPCR was used to determine the presence of human cells in mouse brains. Presented is the percentage of micrometastasis positive mice (black) and the percentage of micrometastasis‐free mice (white) in each group.

The results of the in vitro experiments demonstrated that ALDOC promotes the malignant phenotype of certain human melanoma cells, whereas it exerts opposite effects or no effects on other melanoma cells. We then asked whether ALDOC also functions in an opposite manner in vivo with respect to promoting or inhibiting the malignancy phenotype of melanoma cells. ALDOC overexpressing and control YDFR.CB3, M12.CB3, and DP.CB2 cells were inoculated orthotopically (subcutaneously) into nude mice. Tumors originated from the ALDOC overexpressing M12.CB3 cells were significantly larger than tumors originated from control M12.CB3 cells (Fig. 3A). No significant differences in tumor volume were observed in the mice inoculated with YDFR.CB3 or with DP.CB2 cells.

We next asked whether ALDOC overexpression would influence the capacity of melanoma cells to form spontaneous brain micrometastasis. Nude mice xenografted subcutaneously with ALDOC overexpressing and control YDFR.CB3, M12.CB3, or DP.CB2 cells were sacrificed 52, 59, or 62 days (respectively) postinoculation, and the presence of human melanoma micrometastatic cells in the brains of the xenografted mice was assessed by RT–qPCR using human‐specific gene primers [20]. None of the brains of mice inoculated with control YDFR.CB3 cells contained human cells, while brains of 3 of 8 (37.5%) mice inoculated with ALDOC overexpressing YDFR.CB3 cells contained human cells (Fig. 3B). On the contrary, brains of 5 of 8 (62.5%) mice inoculated with control DP.CB2 cells contained human cells, while brains of 3 of 8 (37.5%) mice inoculated with ALDOC overexpressing DP.CB2 cells contained human cells. No significant difference was observed between control and ALDOC overexpressing M12.CB3 inoculated mice with respect to spontaneous brain metastasis formation.

These results again accentuate intertumor heterogeneity with respect to the functional responses of melanoma cells to ALDOC overexpression. Overexpression of ALDOC augments the metastatic capacity of YDFR.CB3 cells, whereas this enzyme exerts an opposite effect on DP.CB2 cells and no effect on M12.CB3 cells.

### 3.2 ALDOC alters the gene expression profile of melanoma cells

**Fig. 4:**
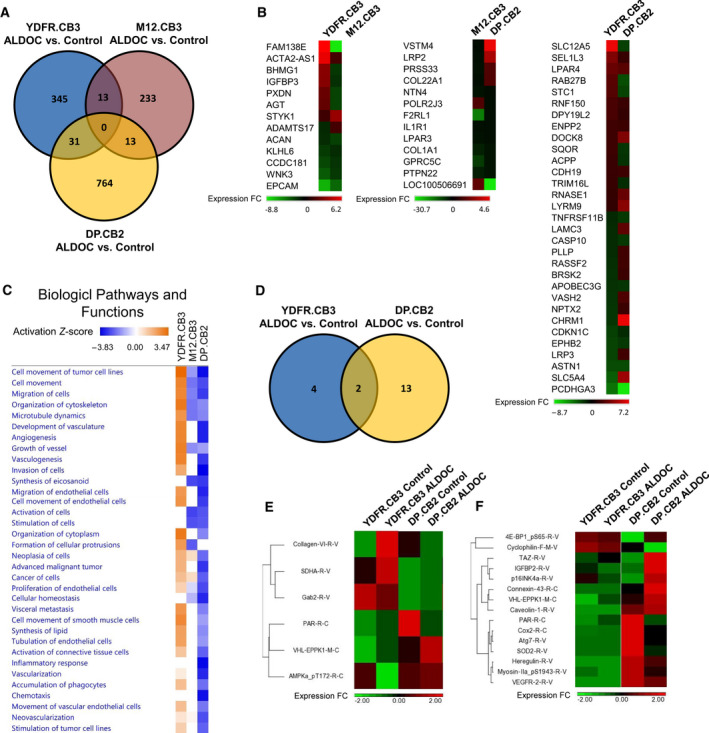
ALDOC alters gene and protein expression in melanoma cells. (A) A Venn diagram demonstrating the number of genes differentially expressed (DE) in ALDOC overexpressing melanoma cells compared with their matching control cells [*P*‐value < 0.05, fold change (FC) ≤ −1.5 or FC ≥ 1.5], as obtained by RNA‐seq analysis. (B) Heat maps of common DE genes whom expression was altered in two melanoma brain metastatic (MBM) cells. (C) Top disease pathways and biological functions enriched for genes significantly regulated following ALDOC overexpression, as obtained by the Ingenuity Pathway Analysis (ipa) software, using comparison analysis. The net effects of gene expression changes in pathway activation or repression were determined using activation *z*‐scores (threshold, *z*‐score < −2 or *z*‐score > 2). Block color intensity corresponds to downregulated (blue) and upregulated (orange) pathways. (D) A Venn diagram demonstrating the number of proteins DE in ALDOC overexpressing MBM cells compared with their matching control cells [fold change (FC) ≤ −1.2 or FC ≥ 1.2], as obtained by RPPA analysis. (E, F) Heat maps of DE proteins in ALDOC overexpressing YDFR.CB3 (E) and DP.CB2 (F) cells.

To identify genomic signatures underlying the heterogeneous functional intertumor response to ALDOC, we profiled gene expression of MBM cells with ALDOC overexpression compared with the respective control cells. RNA‐seq analysis showed that ALDOC overexpression induced differential expression of 389 genes in YDFR.CB3 cells, 259 genes in M12.CB3 cells, and 808 genes in DP.CB2 cells [*P* < 0.05, fold change (FC) ≤ −1.5 or FC ≥ 1.5] compared with corresponding control cells. A Venn diagram and the respective heat map (Fig. 4A,B) indicated that 13 genes were differentially expressed (DE) in both YDFR.CB3 and M12.CB3 ALDOC overexpressing cells, 13 genes were DE in both M12.CB3 and DP.CB2 ALDOC overexpressing cells, and 31 genes were DE in both YDFR.CB3 and DP.CB2 ALDOC overexpressing cells. No common genes were affected in the three different cell line comparisons.

Top disease pathways and biological functions associated with the significantly up‐ or downregulated genes in the three ALDOC overexpressing MBM cells were identified by Ingenuity Pathway Analysis (ipa) (Fig. 4C). Based on this analysis, we observed a distinctive pattern of activation/inhibition of metastasis‐related pathways between the YDFR.CB3 and DP.CB2 cells. Whereas pathways such as cell motility/migration, invasion, organization of cytoskeleton, angiogenesis, and advanced malignant solid tumor were activated in ALDOC overexpressing YDFR.CB3 cells, these same pathways were inhibited in ALDOC overexpressing DP.CB2 cells. The ALDOC overexpressing M12.CB3 cells demonstrated a less definitive phenotype with respect to these pathways. To summarize, the analysis of biological functions and disease pathways in cells with ALDOC overexpression clearly indicated the ALDOC‐mediated induction of genes associated with tumor progression in YDFR.CB3 cells and antitumor progression properties in DP.CB2 cells.

### 3.3 Reverse‐phase protein array analysis of melanoma cells differentially expressing ALDOC

Reverse‐phase protein array analysis [33] was performed in ALDOC overexpressing or control MBM cell lines in order to identify proteins involved in ALDOC‐mediated regulation of melanoma malignancy phenotype. ALDOC overexpressing YDFR.CB3 and DP.CB2 melanoma cells were compared to the corresponding control cells expressing endogenous ALDOC levels. For each pair of cells, we established a list of proteins that were DE in a FC ≤ −1.2 or FC ≥ 1.2. Six proteins were DE in ALDOC overexpressing YDFR.CB3 cells, and fifteen proteins were DE in ALDOC overexpressing DP.CB2 cells, compared with control melanoma cells (Fig. 4D–F). Protease‐activated receptor (PAR) and Epiplakin 1 (EPPK1) were DE in ALDOC overexpressing YDFR.CB3 cells and in DP.CB2 cells. PAR, together with its co‐factors of the hemostatic system, promotes tumor cell migration, angiogenesis, and interactions with cells of the vasculature [34]. EPPK1 is one of the members of the plakins family, which function as cytolinkers connecting elements of the cytoskeleton with each other and to junctional complexes. Plakins are involved in cell proliferation, migration, adhesion, and signal transduction [35].

By integration of RNA‐seq and RPPA analysis, we found that PAR, IGFBP2, Connexin 43, and SOD2 were similarly altered in both assays, emphasizing their significance in the regulatory role of ALDOC of melanoma malignancy.

### 3.4 ALDOC overexpressing melanoma cells may promote the viability of microglia and facilitate their migratory capacity

Various brain pathologies, such as CNS injury or inflammation, are associated with microglia activation and proliferation [4, 36]. As previously reported [4], microglia supports and promotes promalignancy traits such as proteolysis of extracellular matrix (ECM) proteins and migratory capacities in melanoma cells. An increased proliferation of microglia cells and/or recruitment of such cells to the metastatic site, in response to melanoma‐derived cues, may promote melanoma brain metastasis.

**Fig. 5:**
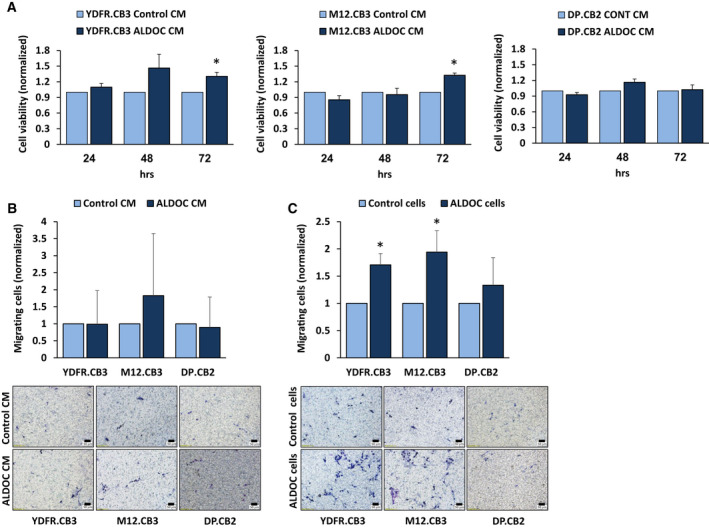
ALDOC overexpressing melanoma cells differently induce changes in microglia phenotype. (A) Microglia cells were cultured for 72 h with conditioned medium (CM) obtained from ALDOC overexpressing or control YDFR.CB3, M12.CB3, and DP.CB2 cells. Cell viability of microglia cells was measured after 24, 48, and 72 h using XTT‐based assay. Absorbance at 450 nm was determined for each well, and subtraction of nonspecific readings (measured at 630 nm) was calculated. The bars represent the average viability of microglia cells treated with CM of ALDOC overexpressing melanoma cells normalized to the viability of microglia cells treated with CM of control melanoma cells at the indicated time point + SEM in at least three independent experiments. Six replicates were performed in each experiment. Significance was evaluated using Student's *t*‐test for microglia cells treated with CM of ALDOC overexpressing melanoma cells, compared with CM of control melanoma cells. *P < 0.05. (B, C) Microglia cells were allowed to migrate through collagen‐coated transwells toward CM obtained from ALDOC overexpressing (ALDOC) or control YDFR.CB3, M12.CB3, and DP.CB2 melanoma cells (B) or toward ALDOC overexpressing or control YDFR.CB3, M12.CB3, and DP.CB2 melanoma cells (C) for 24 h. The migrated cells were fixed and counted in 6–10 fields. The bars represent the average number of microglia cells migrating toward ALDOC overexpressing melanoma CM or cells per field in at least three independent experiments performed in duplicates, normalized to microglia cells migrating toward control melanoma CM or cells (respectively) + SEM. Significance was evaluated using Student's *t*‐test for microglia cells migrating toward ALDOC overexpressing melanoma CM or cells, compared with microglia migrating toward control melanoma CM or cells.*P < 0.05. Representative images are presented (10× magnification, scale bar: 50 µm).

Microglia cell viability was measured following exposure for 24, 48, and 72 h to CM from control or ALDOC overexpressing brain‐metastasizing YDFR.CB3 and M12.CB3 cells. As shown in Fig. 5A, microglia cells treated with CM from ALDOC overexpressing brain‐metastasizing YDFR.CB3 and M12.CB3 cells for 72 h exhibited a significantly higher viability, compared to microglia cells treated with control YDFR.CB3 or M12.CB3 cells. CM of ALDOC overexpressing or of control DP.CB2 cells did not exert any different effect on microglia cell viability.

Recruitment of microglia cells to regions of neuroinflammation and their clustering around metastatic lesions is a crucial event in brain pathologies including brain metastasis [4, 37, 38, 39, 40]. The recruitment of microglia cells to MBM lesion may be induced by a mutual chemo‐attraction. To examine this possibility, we compared the capacity of ALDOC overexpressing and control melanoma cells to attract microglia cells. Soluble factors present in the CM derived from ALDOC overexpressing melanoma cells or control cells attracted microglia cells to the same extent (Fig. 5B). Conversely, the migration of microglia cells toward ALDOC overexpressing YDFR.CB3 and M12.CB3 melanoma cells was significantly enhanced compared with their migration toward control YDFR.CB3 and M12.CB3 melanoma cells (Fig. 5C). In contrast, ALDOC overexpressing DP.CB2 cells did not enhance the migration of microglia toward these melanoma cells.

## 4 Discussion

Advanced‐stage tumors are usually characterized by a high glycolytic rate and genes of the glycolytic pathway are overexpressed in many cancer types including brain cancer [41]. Overexpressed glycolytic enzymes play important roles in cancer progression; they support tumor growth, resistance to cytotoxic therapy, and overall predict poor disease outcome [9, 42].

In addition to its known role in glycolysis, the glycolytic enzyme aldolase C (ALDOC) is involved in several moonlighting, and nonmetabolic functions including cytoskeleton remodeling affecting cell migration via actin filament binding [9].

Previous results from our laboratory [4] demonstrated that following a cross‐talk with microglia, human melanoma cells undergo a functional reprogramming that promotes their malignant phenotype. A prominent manifestation of this reprogramming was an upregulation of ALDOC.

In order to establish whether or not ALDOC plays a role in shaping the malignant phenotype of melanoma cells, we overexpressed or knocked‐down ALDOC expression in three different melanoma brain metastatic (MBM) variants (YDFR.CB3, M12.CB3, and DP.CB2), all of which overexpressed ALDOC following exposure to microglia‐derived factors.

ALDOC overexpression promoted the proliferation of these three melanoma variants when grown in the presence of microglia‐derived factors. This suggested that ALDOC confers a proliferative advantage specifically in the microenvironmental setting of the brain.

Assaying the functional consequences of upregulating ALDOC expression on parameters of tumor progression, we discovered that the three melanoma variants responded differently to alterations in ALDOC expression.

The upregulation of ALDOC expression in YDFR.CB3 cells amplified their malignancy as judged from several in vitro and in vivo functional assays. In sharp contrast, ALDOC overexpression in DP.CB2 cells restricted their malignancy phenotype or did not affect it. M12.CB3 cells expressed intermediate responses to ALDOC overexpression.

These functional data are supported by RNA‐seq analysis. Genes altered in ALDOC overexpressing YDFR.CB3 cells regulate biological pathways that are strongly linked to metastasis formation, including ECM remodeling, cell adhesion, cell migration, regulation of cell proliferation, and integrin binding. For example, IPA upstream regulator analysis identified several activating or suppressive genes that are regulated by ALDOC upregulation. Among them were TNF, NFkB, ERK1/2, IL1B, PDGFBB, JUN, and TGFB1. All these genes were regulated in an opposite manner in ALDOC overexpressing YDFR.CB3 and DP.CB2 cells.

The differential response of YDFR.CB3 and DP.CB2 cells to ALDOC upregulation is a manifestation of intertumor heterogeneity occurring in tumors from different patients but belonging to the same histological type [43]. An important manifestation of intertumor heterogeneity is a diverse response to therapy of such tumors [44]. Intertumor heterogeneity being a recognized significant issue in treatment planning has been evaluated mainly by clustering tumors based on gene expression or RNA sequencing data.

The results of this study if corroborated by testing a large number of melanoma cell lines may provide an additional predictive approach to cluster patients to those who will benefit from a certain drug, to those who will not, and to patients whose condition may worsen by this treatment. Such an approach can be applied by functional assays such as those described in the present study.

Alternatively, or in addition, molecular signatures that have been established by analyzing the functional responses of cells exposed to signals delivered directly or indirectly by chemical or biological drugs may be used for the same purpose.

GM‐CSF‐mediated signaling is a case in point. In a previous study [8], we demonstrated that melanoma‐derived soluble factors stimulated GM‐CSF secretion from BECs and from astrocytes. This cytokine, in turn, boosted the malignancy of cells from one melanoma and diminished the malignancy of another melanoma. In view of the fact that GM‐CSF is used as an adjuvant in various cancer immunotherapy trials (including melanoma) [8], it would be of great benefit to exclude patients bearing tumors whose malignancy may be enhanced by GM‐CSF treatment from this therapy modality.

The finding that ALDOC plays a tumor‐promoting function in certain melanomas should be taken into consideration when planning therapy trials involving targeting of glycolysis pathways [45, 46, 47] or when ALDOC itself is targeted.

The reality of intertumor heterogeneity, caused by a variety of mechanisms [48, 49], underscores the necessity for individualized cancer therapy also in cases where constituents of the tumor microenvironment are targeted.

## 5 Conclusion

This study illuminates hitherto unknown aspects of intertumor heterogeneity. The glycolytic enzyme aldolase C (ALDOC) whose expression in melanoma cells was upregulated by microglia reshapes the malignant phenotype of melanoma cells. The response of melanoma cells from different patients to activation signals delivered by ALDOC varied considerably. In one case, ALDOC intensified the malignancy of the melanoma cells as manifested by an upregulated expression of malignancy‐promoting genes and by enhanced pro malignancy functions in vitro and in vivo. The response of cells from another melanoma patient to ALDOC was diametrically opposed; ALDOC moderated the malignant phenotype of these cells.

These results should be corroborated by analyzing the functional response of cells from a larger cohort of melanoma patients to a variety of activating signals including chemical or biological drugs. If confirmed, this manifestation of intertumor heterogeneity may be developed as an additional method to predict response of tumors to therapy.

## Conflict of interest

The authors declare no conflict of interest.

## Author contributions

SI and IPW conceptualized the data; SI curated the data; MP‐C, MAB, IRR, and DSBH involved in formal analysis; DSBH and IPW acquired funding; SI, SB‐M, OS‐A, SM, AT, and TM investigated the data; SI, SB‐M, and OS‐A designed methodology; OS‐A and IPW administered the project; MP‐C and DSBH provided resources; IPW supervised the data; SB‐M and SM validated the data; SI visualized the data; SI and IPW wrote the original draft; and SI, MAB, IRR, DSBH, and IPW wrote, reviewed, and edited the manuscript. All authors have read and agreed to the published version of the manuscript.
